# Public Perceptions of the Food and Drug Administration’s Proposed Rules Prohibiting Menthol Cigarettes on Twitter: Observational Study

**DOI:** 10.2196/42706

**Published:** 2023-02-10

**Authors:** Runtao Zhou, Qihang Tang, Zidian Xie, Dongmei Li

**Affiliations:** 1 Goergen Institute for Data Science University of Rochester Rochester, NY United States; 2 Department of Clinical and Translational Research University of Rochester Medical Center Rochester, NY United States

**Keywords:** menthol cigarettes, Food and Drug Administration, FDA, FDA's proposed rules, Twitter, perception

## Abstract

**Background:**

On April 28, 2022, the Food and Drug Administration (FDA) proposed rules that prohibited all menthol-flavored cigarettes and other flavored cigars to prevent the initiation of tobacco use in youth and reduce tobacco-related diseases and death.

**Objective:**

The objective of this study was to investigate public perceptions of the FDA’s proposed menthol cigarette rules on Twitter.

**Methods:**

Through the Twitter streaming application programming interface, tobacco-related tweets were collected between April 28, 2022, and May 27, 2022, using a set of keywords, such as smoking, cigarette, and nicotine. Furthermore, 1941 tweets related to the FDA’s proposed menthol cigarette rules were extracted. Based on 300 randomly selected example tweets, the codebook for the attitudes toward the FDA’s proposed rules and related topics was developed by 2 researchers and was used to label the rest of the tweets.

**Results:**

Among tweets related to the FDA’s proposed menthol cigarette rules, 536 (27.61%) showed a positive attitude, 443 (22.82%) had a negative attitude, and 962 (49.56%) had a neutral attitude toward the proposed rules. Social justice (210/536, 39%) and health issues (117/536, 22%) were two major topics in tweets with a positive attitude. For tweets with a negative attitude, alternative tobacco or nicotine products (127/443, 29%) and racial discrimination (84/536, 16%) were two of the most popular topics.

**Conclusions:**

In general, the public had a positive attitude toward the FDA’s proposed menthol cigarette rules. Our study provides important information to the FDA on the public perceptions of the proposed menthol cigarette rules, which will be helpful for future FDA regulations on menthol cigarettes.

## Introduction

About 22.3% (36.7% of men and 7.8% of women) of the world population smoked cigarettes in 2020 [1]. In the United States, 13 of every 100 adults older than 18 years smoke cigarettes [2]. Smoking is the leading cause of preventable death due to tobacco-related diseases [3]. Smokers are exposed to nearly 7000 toxic chemicals [4]. Those chemicals can induce diseases such as cancer, stroke, and emphysema [5-7]. Although there is an overall declining trend in smoking globally, the popularity of menthol cigarettes has increased steadily over the past decade from 31% in 2010 to 37% in 2020 in the domestic cigarette market share [8,9]. In 2021, 39% of middle school and high school students who smoked cigarettes reported using menthol cigarettes [10]. In 2016, the percentage of smokers in African American communities and young adults (aged 18-25 years) who use menthol cigarettes were 85% and 51%, respectively [11]. Almost 9 of 10 African American smokers use menthol cigarettes, compared to less than 30% of White people who smoke [12]. Studies have found that tobacco companies disproportionally marketed menthol cigarettes to African American communities [13-15].

Menthol cigarettes have been marketed and perceived as healthier alternatives to nonmenthol cigarettes [16]. Thus, they attract inexperienced consumers, particularly teenagers, who find nonmenthol cigarettes undesirable [17]. Numerous studies have shown that menthol cigarettes not only cause respiratory diseases and cancer just like nonmenthol cigarettes do but also produce worse cardiovascular effects compared to nonmenthol cigarettes [18-20]. One study indicated that the removal of menthol from cigarettes can help prevent youth from smoking as well as help the cessation of smoking in adults [18]. To prevent the initiation of smoking in youth and to reduce tobacco-related diseases, on April 28, 2022, the FDA proposed rules to regulate menthol cigarettes and all flavored cigars except for tobacco-flavored cigars [21]. Knowing about the public perceptions and discussions of the proposed menthol cigarette rules could provide important information to the FDA for their future regulations of menthol cigarettes.

Over the years, Twitter has become one of the most popular and influential social media platforms [22]. Twitter provides people with opportunities to share their own opinions about government policies [23]. Besides the public comments the FDA has collected, Twitter provides an extra channel to learn the public perceptions and discussions about the proposed rules on menthol cigarettes in real time [24]. Many recent studies have used Twitter data to study public perceptions of different government policies related to tobacco products, such as the FDA and New York State’s flavor policy or regulation [25,26]. In this study, we aimed to study public perceptions and discussions about the FDA’s proposed menthol cigarette rules on Twitter. Our study provides the FDA with timely information on public perceptions of the FDA’s proposed menthol cigarette rules on Twitter.

## Methods

### Data Collection and Processing

Through Twitter streaming application programming interface, we collected Twitter posts (tweets) related to tobacco or nicotine between April 28th, 2022, and May 27th, 2022, using a set of keywords, including “smok*,” “cig*,” “nicotine,” and “tobacco” [27,28]. After we removed duplicate tweets and retweets, there were 323,444 tweets related to tobacco and nicotine in the data set.

### Data Processing

Using the hashtag “mentholban,” some sample tweets related to the FDA’s proposed menthol cigarette rules can be directly identified on Twitter. After finding some common keywords among the sample tweets, our final keyword list includes the following: “ban & menthol,” “ban & mentholcigarette,” “prohibit & menthol,” “prohibit & mentholcigarette,” “get rid of & menthol,” “get rid of & mentholcigarette,” “fda & ban,” “fda &prohibit,” “fda & get rid of,” “fda & prohibit,” “fda & rule,” and “fda & policy.” The tense of the verbs, the plural form of the nouns, and the different names of the FDA were all considered. Through further filtering with the keywords from the keyword list, 1989 tweets that could be related to the FDA’s proposed menthol cigarette rules were identified.

### Content Analysis

From 1989 tweets that were potentially related to the FDA’s proposed menthol cigarette rules, 300 samples were randomly selected and manually coded individually by 2 researchers. Firstly, each tweet was manually determined if it was relevant to the FDA’s proposed menthol cigarette rules. Then, each relevant tweet was checked for the attitude toward the FDA’s proposed menthol cigarette rules as either positive, negative, or neutral. A positive attitude means that the corresponding tweets generally supported the FDA’s proposed menthol cigarette rules, a negative attitude means that the corresponding tweets generally disagreed with the FDA’s proposed menthol cigarette rules, and a neutral attitude means that the tweets did not show any clear attitude. Lastly, each tweet was grouped into different topics based on its content. Tweets that had a positive sentiment toward the FDA’s proposed menthol cigarette rules were categorized into the positive attitude category. Normally, those tweets contained the keywords “support,” “agree,” and “like.” On the other hand, tweets that had negative sentiments toward the FDA’s proposed menthol cigarette rules were categorized into the negative attitude category. Keywords such as “dislike,” “disagree,” and “shouldn’t” helped identify those tweets that conveyed negative attitudes. Tweets in which it was hard to identify the sentiments toward the FDA’s proposed menthol cigarette rules were categorized into the neutral attitude category. Those tweets, such as “The U.S. government on Thursday will lay out its long-awaited plan to ban menthol cigarettes…,” were typically about news articles or other information not directly related to the FDA’s proposed menthol cigarette rules. Tweets with a positive attitude were further categorized into 6 different topics, primarily “preventing smoking-related diseases” (discussions of how the proposed menthol regulations can be beneficial to smokers with physical health conditions) and “reducing disproportionate tobacco use among minorities” (discussions of how African American communities have been disproportionately targeted by the advertisement of menthol cigarettes), and other topics including “preventing youth addiction” and “preventing nicotine addiction.” Tweets with a negative attitude were categorized into 7 topics, primarily “ignoring alternative tobacco/nicotine products” (discussions of the futility of the proposed regulation due to the existence of many other alternative products with nicotine) and “discriminating minority groups” (discussions of the proposed regulation discriminating against African American smokers who are the major users of menthol cigarettes). Other topics included “impeding the freedom to smoke,” “having potential enforcement problems,” and “hindering needed nicotine intake.” For the rest of the tweets that displayed either positive or negative attitudes, they were classified into “others” if their topics were not representative enough to become a separate category, and they were classified into “no reasons” if they only expressed attitudes toward the proposed regulation but did not mention any specific reason. A more detailed description of different topics in positive and negative attitudes with sample tweets for each topic is shown in Table S1 in Multimedia Appendix 1. The Kappa statistic for interrater reliabilities between the 2 researchers was 0.91 based on the selected 300 samples. Any discrepancy was resolved by a group of 4 members.

With the development of a codebook for hand coding, the rest of the tweets related to the FDA’s proposed menthol cigarette rules were coded individually by the 2 researchers. Each tweet was assigned only to 1 topic.

### Ethics Approval

This study was reviewed and approved by the Office for Human Subject Protection Research Subjects Review Board at the University of Rochester (Study00006570).

## Results

Among the 1989 tweets that might have been related to the FDA’s proposed mental cigarette rules, 48 tweets contained the relevant keywords but were not related to the proposed rules on menthol cigarettes, and 1941 tweets were relevant to the FDA’s proposed menthol cigarette rules. Among 1941 relevant tweets, 536 (27.61%) had a positive attitude, 443 (22.82%) had a negative attitude, and 962 (49.56%) had a neutral attitude toward the FDA’s proposed menthol cigarette rules.

Among the positive-attitude tweets, the most prevalent topic was “reducing disproportionate tobacco use among minorities” (210/536, 39%), followed by “preventing smoking-related diseases” (117/536, 22%), “preventing youth addiction” (84/536, 16%), “preventing nicotine addiction” (19/536, 2%), and “others” (13/536, 2%; Table 1). In addition, 102 (19%) tweets in “no reason” category did not provide any reason. Specifically, for “reducing disproportionate tobacco use among minorities,” many tweets (eg, “Menthol cigarettes and flavored cigars, have been used to lure countless Black Americans—into lifelong nicotine addiction…”) argued that big tobaccos had disproportionately targeted African American communities with menthol cigarette advertisements, caused their long-term reliance on nicotine and a huge health disparity. For “preventing smoking-related diseases,” many tweets, such as one mentioning “Today’s decision…to ban menthol-flavored cigarettes is one of the most consequential to improve public health in decades, especially considering that tobacco smoking remains a leading killer even after all these years,” argued that the proposed regulation is a step further in tobacco control, helps prevent smoking-related diseases, and improves the public health. The positive-attitude tweets in general support the proposed menthol cigarette rules due to the potential benefits to not only the public but also some minority groups.

Among the negative-attitude tweets, the most popular topic was “ignoring alternative tobacco/nicotine products” (127/443, 29%), followed by “others” (105/443, 24%), “discriminating minority groups” (70/443, 16%), “impeding the freedom to smoke” (49/443, 11%), “having potential enforcement problems” (47/443, 11%), “no reason” (27/443, 6%), and “hindering needed nicotine intake” (18/443, 4%). For “ignoring alternative tobacco/nicotine products,” most of the tweets, such as “FDA to issue plan banning menthol in cigarettes, cigars #SmartNews (won’t folks just switch to non-menthol? It’s the addiction of nicotine not menthol),” expressed concerns about the effectiveness of the proposed regulation, given the various alternative products containing nicotine to which the current menthol cigarette smokers can switch. Others also raised concerns over the targets of the regulation. For “discriminating minority groups,” many tweets, such as the one that follows, argued that the regulation is racial discriminative given that the majority of smokers from the African American communities smoked menthol cigarettes:

If that’s what they care about, ban all cigarettes. But it’s not. They want another excuse to pin crimes on black people. 85% of black smokers smoke menthols, this is clearly targeted at them.

The tweets with a negative attitude questioned the FDA’s proposed menthol cigarette rules from several angles, from the effectiveness of the rules, and the targeted groups, to even the feasibility of the enforcement.

**Table 1:** Topics for the proposed menthol cigarette rules–related tweets with either a positive or negative attitude.

Tweets and topics			Values, n (%)
**Positive attitude (n=536)**			
	Reducing disproportionate tobacco use among minorities	210 (39)	
	Preventing smoking-related diseases	117 (22)	
	Preventing youth addiction	84 (16)	
	Preventing nicotine addiction	10 (2)	
	No reason	102 (19)	
	Others	13 (2)	
**Negative attitude (n=443)**			
	Ignoring alternative tobacco/nicotine products	127 (29)	
	Discriminating minority groups	70 (16)	
	Impeding the freedom to smoke	49 (11)	
	Having potential enforcement problems	47 (11)	
	Hindering needed nicotine intake	18 (4)	
	No reason	27 (6)	
	Others	105 (24)	

## Discussion

### Principal Findings

In this study, we investigated the public perceptions and discussions of the FDA’s proposed menthol cigarette rules using collected Twitter data from April 28, 2022, to May 27, 2022. Overall, we identified a more positive attitude toward the FDA’s proposed menthol cigarette rules during our study period. This result indicated that there was more support in the public for the FDA’s proposed menthol cigarette rules. The main topics for tweets with a positive attitude included “reducing disproportionate tobacco use among minorities” and “preventing smoking-related diseases.” Among tweets with a negative attitude, “ignoring alternative tobacco/nicotine products” and “discriminating minority groups” were the top two topics. Prohibiting menthol cigarettes could benefit the African American community by potentially reducing their smoking prevalence and improving their health, while the availability of many other tobacco or nicotine products on the market could diminish the potential benefits from the FDA’s proposed menthol cigarette rules. In addition, the high prevalence of menthol cigarette usage in the African American community also led to potential racial discrimination issues. Our study results provided important information on the public perceptions and discussions about the FDA’s proposed menthol cigarette rules, which will be valuable for FDA’s future actions on menthol cigarettes.

### Comparison With Prior Work

Although the regulation was proposed in April 2022, the discussions of the restriction of menthol cigarettes have appeared since the FDA banned other characterizing flavors in cigarettes under the 2009 Family Smoking Prevention and Tobacco Control Act [29]. A previous study has analyzed the impact of either hypothetical or implemented menthol cigarette regulations, and one previous study has summarized these analyses and concluded that banning menthol cigarettes could assist with smoking cessation and lower smoking initiation [30]. Nevertheless, these studies analyzed the behaviors of individual smokers and did not consider the reactions of the public. Our study instead focused on the reactions of the public to the FDA’s proposed menthol cigarette rules on Twitter after the proposition of the rules. The support and concerns of the public would be helpful to the FDA if the proposed menthol cigarette rules will be implemented in the future.

In our study, slightly more users showed positive attitudes toward the FDA’s proposed menthol cigarette rules. “Reducing disproportionate tobacco use among minorities” was the most frequently discussed topic among the supporters. One previous publication has also argued that both the disproportionate menthol cigarette advertisement and the exemption of menthol flavor from the 2009 Family Smoking Prevention and Tobacco Control Act are the results of social injustice experienced by minority communities [31]. Another similar publication has discussed how a menthol cigarette ban can protect African American communities [32]. In the real world, many community-based organizations in African American communities were also taking action to educate members of their communities about the risks of smoking menthol cigarettes and engaging community members to reduce the use of menthol cigarette [33]. The proposed menthol cigarette rules will further support community-based organizations’ efforts in reducing menthol cigarette smoking among African Americans.

Two other popular reasons supporting the proposed menthol cigarette rules on Twitter are the concerns over the overall health effect of menthol cigarettes and the potential of such rules to prevent youth from nicotine addiction. Although menthol cigarette is not generally more harmful than nonmenthol cigarettes, being biomedically active, menthol may interact with nicotine and 4-(methylnitrosamino)-1-(3-pyridyl)-1-butanol, which can damage cells and reduce the feelings of respiratory discomfort [34]. In addition, menthol cigarettes contribute to both the initiation and the addiction of smoking cigarettes among youth [35]. The FDA’s Tobacco Products Scientific Advisory Committee also concluded that more children began smoking menthol cigarettes, and they were more likely to be addicted and become long-term users [36]. As a result, a regulation on menthol cigarette products seems to be beneficial, especially for African American communities. However, other concerns over the proposed regulation should not be ignored.

Among tweets with a negative attitude, the most popular topic was “ignoring alternative tobacco/nicotine products.” These tweets argued that nicotine is the main factor that causes addiction. One of them stated that such a regulation would be futile because people would still use other nonmenthol cigarette products that contain nicotine like e-cigarettes. A study on the impact of a comprehensive tobacco product flavor ban in San Francisco found that the tobacco products flavor ban cannot immediately reduce the use of flavored tobacco products individually; although it can significantly reduce the overall e-cigarette and cigar use, it may at the same time increase cigarette use [37]. The proposed national menthol cigarette rules may have different effects in comparison to a local flavor ban; however, the potential problems, such as the existence of alternative tobacco products, might diminish the effectiveness of the menthol cigarette rules. Several studies have also examined the reaction of menthol cigarette smokers toward the FDA’s previous menthol cigarette regulations [38-40]. The impacts of those regulations seem to correlate well with the availability of e-cigarettes, which could be an alternative product to menthol cigarettes. These studies have found that around 15%-30% of the current menthol cigarette smokers would switch to e-cigarettes if menthol cigarettes were banned [38-40].

Our study has several limitations. The demographic distributions of Twitter users are slightly different from the general population in the United States. For example, a large proportion (38.5%) of Twitter users are between the ages of 25 and 34 years [41], which is much higher than the proportion of adults aged 25-34 years (13.7%) in the United States [42]. Thus, the result we obtained from this study will not fully represent the perceptions of the FDA’s proposed menthol cigarette rules in the general US population. Furthermore, the limited number of keywords used in this study could make us miss some tweets and introduce some biases. Similarly, due to the limited access to demographical information of Twitter users, we cannot determine if Twitter users with different demographics had different perceptions of the proposed rules on menthol cigarettes.

### Conclusions

Generally, the public held a favorable view toward the FDA’s proposed menthol cigarette rules. Analyzing public perceptions of the FDA’s proposed menthol cigarette rules on Twitter provided important information for the FDA before either the enforcement or the revision of the proposed menthol cigarette rules.

## Appendix

Multimedia Appendix 1 Codebook for hand coding tweets related to the menthol cigarette ban.

## Abbreviations

FDA: Food and Drug Administration

## Data Availability

Data are available upon reasonable request from the corresponding author.
