# Supplementary material for: Public Perceptions of the Food and Drug Administration’s Proposed Rules Prohibiting Menthol Cigarettes on Twitter: Observational Study
Source: JMIR Form Res. 2023 Feb 10;7:e42706. doi: 10.2196/42706 (PMC9960014; doi:10.2196/42706)
Supplement: Multimedia Appendix 1 [file formative_v7i1e42706_app1.docx]

**Table S1. Codebook for hand-coding tweets related to the FDA’s proposed menthol cigarette rules.**

| **Attitude** | **Topics** | **Description** | **Example Tweets** |
| --- | --- | --- | --- |
| **Positive** | **Preventing Smoking-related Diseases** | Discussing how the menthol cigarette regulation will reduce the occurrence of major causes of death, such as heart disease, stroke, cancer, and diabetes, among smokers. | “There’s no question that a menthol cigarette ban will save many Black lives. Tobacco is a major contributor to the four leading causes of death among African Americans: heart disease, cancer, stroke, and diabetes.  ” |
|  | **Preventing Youth Addiction** | Discussing how menthol cigarettes can make it easier for youth to initiate tobacco use and how menthol cigarette regulation helps prevent children and teenagers (the next generation) from smoking. | “Fruit and spice flavorings in cigarettes because of their potential appeal to children but left unsettled the question of menthol. The proposed ban has important ramifications for smokers and for tobacco companies.” |
|  | **Preventing Nicotine Addiction** | Discussing how menthol makes nicotine more addictive and how the menthol cigarette regulation helps prevent such addiction. | “… Menthol is an additive that prolongs addictions. Get rid of it ASAP …” |
|  | **Reducing Disproportionate Tobacco Use among Minorities** | Discussing social justice issues, such as how big tobacco companies targeted menthol flavor cigarettes to minority groups, especially African Americans and how menthol cigarette regulation will prevent big tobacco company exploiting African American smokers. | "Menthol cigarettes and flavored cigars have been used to lure countless Black Americans – into lifelong nicotine addiction.  The @US_FDA just proposed ending their sale nationwide. Good.  But Big Tobacco will fight and delay this. Here’s our plan to #DefeatBigTobacco." |
|  | **Others** | Other discussions that do not fall into the categories above and the number of similar tweets is too small to be categorized into an individual topic category. | “Well if for example for me, my goal would be an out right ban of tobacco or, at least cigarettes. So banning menthol is a step in that direction.” |
|  | **No Reason** | Showing only positive sentiments with no obvious reasons. | “FDA finally moving forward to ban menthol tobacco products. 20+ years too late, but it needs to happen!” |
| **Negative** | **Discriminating Minority Groups** | Arguing about that since the majority of menthol cigarette users are people with color, banning the menthol cigarette will harm the interest of minority groups. Most the tweets fall into this category also argue that the FDA should regulate all types of cigarettes instead of just menthol cigarettes. | “lol banning specifically menthol cigarettes but not the other ones that white people smoke” |
|  | **Impeding the Freedom to Smoke** | Arguing about that people have the freedom to choose to smoke menthol cigarettes. Thus, the menthol cigarette regulations essentially take away smokers’ freedom of choice. | “Tell them about the REAL STUDY that people who smoke menthol SMOKE LESS and when it comes to the so called addictive part its no more than any other tobacco! what is this really about TAKING AWAY OUR FREEDOM OF CHOICE” |
|  | **Ignoring Alternative Tobacco/Nicotine Product** | Questioning the target of the menthol cigarette regulation because of the existence of alternative products. The alternative tobacco/nicotine products may either be other tobacco products or other nicotine products that have stronger negative effects. | “Just think FDA/CTP could have save millions of lives by encouraging people to switch to vaping to help people quit.. instead they will just switch to non menthol tobacco …” |
|  | **Having Potential Enforcement Problems** | Questioning the effectiveness of the policy, the logic of the policy. Those tweets normally expressive the mistrust of the FDA. | “The tobacco lobby is really strong. I'm amazed our corrupt politicians are even trying the menthol ban.” |
|  | **Hindering Needed Nicotine Intake** | Arguing about people’s need for menthol cigarettes due to mental reliance, primarily due to the intake of nicotine to relief depression. | “Of course the menthol ban is just precursor to a tobacco ban, which is great except for everyone except those of us out here self medicating for depression bc now we’ll have to take other drugs that are also harmful and not as effective.” |
|  | **Others** | Other discussions that do not fall into the categories above and the number of similar tweets is too small to be categorized into an individual topic category. | "Americans are struggling to find housing and lack access to healthcare due to regulatory restrictions*  FDA: Let’s just ban flavored nicotine…" |
|  | **No Reason** | Discussions that show only negative sentiments with no obvious reasons. | “Government: To stop the government from banning Menthol cigarettes and tobacco - Sign the Petition!” |
| **Neutral** | Discussions that do not display any sentiment or do not display clear sentiments, such as news and general discussions about the policy. | | “FDA issues plan to ban menthol in cigarettes, cigars #SmartNews” |
